# *Mycobacterium chelonei* Breast Abscess Associated With Nipple Piercing

**DOI:** 10.1155/S1064744995000433

**Published:** 1995

**Authors:** Mark D. Pearlman

**Affiliations:** Department of Obstetrics and Gynecology University of Michigan Medical Center 1500 E. Medical Center Dr., D2202 MPB Ann Arbor MI 48109-0718. USA

## Abstract

*Background:* Breast abscesses are typically seen in the setting of complicated mastitis in lactating women. Abscesses resulting from foreign bodies are not commonly seen in the breast. Over the past few decades, body piercing has become increasingly common, yet the infectious morbidity resulting from it is not well recognized. A breast abscess associated with nipple piercing is described in this report.

*Case:* A 19-year-old woman developed a breast abscess approximately 10 weeks after the placement of a nipple ring. Multiple drainage procedures were performed before the infection was controlled. The cultures grew *Mycobacterium chelonei*, an organism not commonly causing soft-tissue infection.

*Conclusion:* Body piercing may be associated with significant infections. The use of sterile equipment and techniques should be used to prevent these uncommon infections.


Infectious Diseases in Obstetrics and Gynecology 3:116–118 (1995)
© 1995 Wiley-Liss, Inc.

### *Mycobacterium chelonei* Breast Abscess Associated With Nipple Piercing

**Mark D. Pearlman**

*Department of Obstetrics and Gynecology, University of Michigan Medical Center, Ann Arbor, MI*

ABSTRACT

***Background:*** **Breast abscesses are typically seen in the setting of complicated mastitis in lactating women. Abscesses resulting from foreign bodies are not commonly seen in the breast. Over the past few decades, body piercing has become increasingly common, yet the infectious morbidity resulting from it is not well recognized. A breast abscess associated with nipple piercing is described in this report.**

***Case:*** **A 19-year-old woman developed a breast abscess approximately 10 weeks after the placement of a nipple ring. Multiple drainage procedures were performed before the infection was controlled. The cultures grew *Mycobacterium chelonei*, an organism not commonly causing soft-tissue infection.**

***Conclusion:*** **Body piercing may be associated with significant infections. The use of sterile equipment and techniques should be used to prevent these uncommon infections.** © 1995 Wiley-Liss, Inc.

KEY WORDS

Nipple ring, mastitis, foreign body

Ear piercing has been a practice for many years among women and, more recently, among men. The infectious morbidity from ear piercing, though difficult to document in the community, appears to be fairly low. Piercing of other body parts has become more popular in the last two decades. Nipple piercing is now a widely practiced procedure that is typically performed outside the medical setting. This procedure is performed with a variety of piercing instruments including sewing needles, safety pins, straight pins, hypodermic needles, and piercing needles. While most "operators" use disposable instruments, some use reusable piercing needles and autoclave their instruments or clean them in other ways. The infectious morbidity of this procedure is unknown in most of these settings, e.g., tattoo parlors, as most states have no regulatory agencies to track these complications. We describe an unusual case of a delayed abscess formation with *Mycobacterium chelonei* following nipple piercing.

### CASE REPORT

A 19-year-old female college student presented to the University Health Service at the University of Michigan with the chief complaints of soreness and swelling in her right nipple. She stated that, approximately 10 weeks prior, she had gone to a local tattoo parlor and had bilateral nipple rings placed. She noted no complications until several days before her presentation to the clinic. On her initial examination, her temperature was 36.8°C, BP was 100/74, and pulse was 72. There was a round, 4-cm area of erythema over the lateral aspect of the right areolar region. Deep to the erythema was a 2-×-3-cm, palpable, fluctuant area. Three milliliters of purulent material was aspirated and sent for aerobic culture and Gram's stain. She denied any

**Address correspondence/reprint requests to Dr. Mark D. Pearlman, Department of Obstetrics and Gynecology, University of Michigan Medical Center, 1500 E. Medical Center Dr., D2202 MPB, Ann Arbor, MI 48109-0718.**

Received February 8, 1995
Accepted July 17, 1995

other trauma to the area. She was started on cephalexin, 250 mg q.i.d. for 10 days. On her repeat examination 3 days later, no improvement was noted in the erythema. The fluctuant area was larger, measuring approximately 3 × 4 cm, without demonstrable axillary adenopathy. She was referred to the University of Michigan Breast Care Center. On her examination there, her oral temperature was 37.1°C, BP was 104/68, and pulse was 76. The right areola was elevated with a region of erythema encompassing approximately 4 cm. Palpation of the breast revealed a single area of fluctuance measuring about 4 × 4 cm with an area of surrounding induration measuring a total of 8 cm. There was no associated axillary, supraclavicular, or intercostal adenopathy appreciated. An HIV test was negative, and no evidence of immunodeficiency was noted.

She was taken to the operating room where a circumareolar incision was made under general anesthesia and a multiloculated abscess was drained of approximately 15 ml of nonfoul-smelling, purulent material. In addition, the wall of the abscess was debrided. The purulent material was sent for aerobic and anaerobic studies and Gram's stain. The Gram's stain revealed multiple WBCs, without organisms seen. The culture grew rare quantities of *Staphylococcus epidermidis*. The wound was packed with 2.54-cm plain gauze and left open. The patient was placed on oral amoxicillin/clavulinic acid, 500 mg t.i.d., and instructed to pack the wound twice daily, irrigating with 0.25% acetic acid. The patient was seen 5 days following her surgery, at which time increased induration of the wound edges was noted. An area of induration without fluctuance approximately 3 cm lateral to the wound was also noted. The amoxicillin/clavulinic acid was stopped and she was started on oral ciprofloxacin, 250 mg b.i.d. She presented again 4 days later complaining of increasing right breast pain. On her examination, she was afebrile with normal vital signs. However, the original wound edges had closed with an underlying 3-×-3 cm area of fluctuance. The previously noted satellite area of induration was further indurated; however, no obvious fluctuance was evident on physical examination or by ultrasound. The original wound was opened under IV sedation and repacked. The ciprofloxacin and twice-daily packing were continued. Six days later, she presented for a scheduled visit, at which time the primary wound remained open, and somewhat indurated, but the satellite wound had obvious fluctuance. She was taken back to the operating room for exploration of the original abscess cavity. A dark, 3-mm lesion was seen on the wall of the cavity facing the satellite lesion. A dissection of this dark lesion with a fine wire probe revealed it to be a sinus tract connecting the original abscess cavity with the satellite cavity. A separate incision was made in the skin overlying the satellite lesion, and both wounds were drained and packed. A #1 Prolene suture was placed along the sinus tract and tied loosely to prevent premature closure of the tract. Aerobic and anaerobic as well as acid-fast bacteria (AFB) stains and cultures were obtained from the fluid and tissue of the abscess wall. The initial AFB stain was positive, and the patient was switched to clarithromycin, 500 mg b.i.d. A subsequent culture revealed *M. chelonei*, which was sensitive to clarithromycin. The wound gradually granulated in with local wound care over the subsequent 4 weeks. The clarithromycin was discontinued after a total of 6 weeks. Six months after her initial presentation, she was doing well without evidence of infection.

### DISCUSSION

*M.chelonei* has been documented in bronchopulmonary infections, as well as in the eye, cardiac valve, bone, retroperitoneum, and soft tissue.[1–6] Skin and soft-tissue infections have been the most frequently documented, although descriptions of breast infections due to this organism could not be found.[7] Typically, soft-tissue infections with *M. chelonei* cause an acute inflammatory reaction with suppuration, but other clinical manifestations including sinus-tract formation and indolent chronic inflammation have been described.[8] Recently, infections have been described in immunocompromised patients, a group that appears to be at risk for disseminated disease.[7,8] In our case, the nipple piercing provided a portal for infection of the duct system.

Breast infection complicating nipple piercing appears to be an extremely rare or under-reported complication, with no cases identified on Medline using numerous search criteria. This case is even more unusual because of the isolation of *M. chelonei*, a fast-growing mycobacterium that can occasionally cause soft-tissue infection. The delay from the placement of the nipple ring until the onset of symptoms (approximately 10 weeks) probably occurred because this organism grows much more slowly than organisms typically causing soft-tissue infection. For example, the primary isolation of *M. chelonei* takes 2–30 days. This fact may also account for delay in the identification of the organism because the culture plates are typically discarded after 7 days of no growth.

*M. chelonei* can frequently be recovered from tap water, soil, dust, and moist areas in the hospital environment. Many bronchopulmonary infections with *M. chelonei* are a result of contamination from the water used to disinfect bronchoscopes.[3] Most human infections are acquired by inoculation after accidental trauma, surgery, or injection. It is possible that the breast infection described here resulted from contamination of the piercing equipment, perhaps from the tap water used to clean or rinse the instrument.

The treatment of these mycobacterial infections involves the surgical removal of all infected tissue in addition to the use of antibacterial agents active against this organism. Amikacin is the most predictably active agent, but many others have reasonably good activity, including clarithromycin, other aminoglycosides, doxycycline, rifampin, and ciprofloxacin.[9,10] Because of the slow growth of the organism, the duration of treatment with antibacterials should be at least 4–6 weeks after a clinical response. The prevention of this type of soft-tissue infection is best achieved through the use of sterile gloves, drapes, appropriate preparation of the skin with an agent such as Betadine, and disposable piercing equipment.

### REFERENCES

1. Idemoyer V, Cherubin CE. Retroperitoneal abscess caused by *Mycobacterium chelonei* and treatment. Ann Pharmacother 27:178–179, 1993.
2. Bullington RH Jr, Lanier JD, Font RL: Nontuberculous mycobacterial keratitis. Report of two cases and review of the literature. Arch Ophthalmol 110:519–524, 1992.
3. Gubler JG, Salfinger M, von Graevenitz A: Pseudoepidemic of nontuberculous mycobacteria due to a contaminated bronchoscope cleaning machine. Report of an outbreak and review of the literature. Chest 101:1245–1249, 1992.
4. Franck N, Cabie A, Villette B, et al.: Treatment of *Mycobacterium chelonei*-induced skin infection with clarithromycin. J Am Acad Dermatol 28:1019–1021, 1993.
5. Pruitt TC, Hughes LO, Blasier RD, et al.: Atypical mycobacterial vertebral osteomyelitis in a steroid-dependent adolescent. A case report. Spine 18:2553–2555, 1993.
6. Repath F, Seabury JG, Sanders CV, et al.: Prosthetic valve endocarditis due to *Mycobacterium chelonei*. South Med J 69:1244–1246, 1976.
7. Wallace RJ Jr, Brown BA, Onyi GO: Skin, soft tissue and bone infections due to *Mycobacterium chelonei:* Importance of prior corticosteroid therapy, frequency of disseminated infections, and resistance to oral antimicrobials other than clarithromycin. J Infect Dis 166:405–412, 1992.
8. Ingram CW, Tanner DC, Durack DT, et al.: Disseminated infection with rapidly growing mycobacteria. Clin Infect Dis 16:463–471, 1993.
9. Brown BA, Wallace RJ Jr, Onyi GO, et al.: Activities of four macrolides, including clarithromycin, against *Mycobacterium fortuitum*, *Mycobaterium chelonei* and *M. chelonei*-like organisms. Antimicrob Agents Chemother 36: 180–184, 1992.
10. Wallace RJ Jr, Swanson JM, Silcox VA, et al.: Treatment of nonpulmonary infections due to *Mycobacterium fortuitum* and *Mycobacterium chelonei* on the basis of in vitro susceptibilities. J Infect Dis 152:500–514, 1985.
